# [Retracted] Aberrant expression of miR-130a-3p in ankylosing spondylitis and its role in regulating T-cell survival 

**DOI:** 10.3892/mmr.2026.13991

**Published:** 2026-08-17

**Authors:** Fengju Li, Dingran Si, Xuejun Guo, Ningru Guo, Dandan Li, Liujing Zhang, Xianan Jian, Jiasheng Ma

Mol Med Rep 20: 3388–3394, 2019; DOI: 10.3892/mmr.2019.10573

Following the publication of the above paper, it was drawn to the Editor's attention by a concerned reader that the expression of BAX protein in Jurkat T cells, as shown in the western blots in Fig. 6A on p. 3392, was unexpected, since Jurkat cells have been reported to be BAX-null [see the paper “A genome-wide survey of mutations in the Jurkat cell line” by Gioia *et al*, Vol. 19, article no. 334 (2018)]. Furthermore, upon performing an independent analysis of the data in this paper, it came to light that the control β-actin western blots featured in Fig. 4A and flow cytometric data included in Fig. 5B were strikingly similar to data that had already been submitted for publication to other journals in other articles written by different authors at different research institutes, one of which has since been retracted.

Owing to the fact that the contentious data in the above article had already been submitted for publication prior to its submission to *Molecular Medicine Reports*, the Editor has decided that this paper should be retracted from the Journal. The authors were asked for an explanation to account for these concerns, but the Editorial Office did not receive a reply. The Editor apologizes to the readership for any inconvenience caused.

